# Long-term medical treatment in congenital hyperinsulinism: a descriptive analysis in a large cohort of patients from different clinical centers

**DOI:** 10.1186/s13023-015-0367-x

**Published:** 2015-11-25

**Authors:** Alena Welters, Christian Lerch, Sebastian Kummer, Jan Marquard, Burak Salgin, Ertan Mayatepek, Thomas Meissner

**Affiliations:** Department of General Pediatrics, Neonatology and Pediatric Cardiology, University Children’s Hospital Duesseldorff, Moorenstrasse 5, Duesseldorf, D-40225 Germany; Cochrane Metabolic and Endocrine Disorders Group, Institute of General Practice, Duesseldorf University Hospital, Duesseldorf, Germany; Department of Pediatric Kidney, Liver and Metabolic Diseases, Hannover Medical School, Hannover Medical School, Germany; Neonatal Intensive Care Unit, Cambridge University Hospitals NHS Foundation Trust, Cambridge, UK; University Department of Obstetrics & Gynaecology, University of Cambridge, Cambridge, UK

**Keywords:** Congenital hyperinsulinism, Persistent hyperinsulinemic hypoglycemia of infancy, Diazoxide, Octreotide, Lanreotide, Nifedipine, Glucagon

## Abstract

**Background:**

Up to now, only limited data on long-term medical treatment in congenital hyperinsulinism (CHI) is available. Moreover, most of the drugs used in CHI are therefore not approved. We aimed to assemble more objective information on medical treatment in CHI with regard to type and duration, dosage as well as side effects.

**Methods:**

We searched MEDLINE (from 1947) and EMBASE (from 1988) using the OVID interface for relevant data to evaluate medical treatment in a large cohort of patients with CHI from different clinical centers. Randomized, controlled trials were not available. We evaluated case reports and case series. No language restrictions were made.

**Results:**

A total number of 619 patients were medically treated and information regarding conservative treatment was available. Drugs used were diazoxide (in 84 % of patients), somatostatin analogues (16 %), calcium channel antagonists (4 %) and glucagon (1 %). Mean dose of diazoxide was 12.5 (±4.3) mg/kg ⋅ d (range 2–60 mg/kg ⋅ d), mean duration of diazoxide treatment until remission was 57 months. Side effects of diazoxide were usually not severe. The causal relation between diazoxide and severe side effects, e.g. heart failure (3.7 %) remains doubtful. Mean dose of octreotide was 14.9 (±7.5) μg/kg ⋅ d (range 2.3–50 μg/kg ⋅ d), of lanreotide 67.3 (±39.8) mg ⋅ month (range 10–120 mg ⋅ month). Mean duration of treatment with somatostatin analogues until remission was 49 months. Frequent side effects included tachyphylaxis and mild gastrointestinal symptoms. The risk of persistent growth deceleration was low (<5 %).

**Conclusions:**

Severe side effects are rare and a causal relation remains disputable. We conclude that long-term conservative treatment of CHI is feasible.

## Background

Congenital hyperinsulinism (CHI) is a heterogeneous disorder leading to increased, often unregulated secretion of insulin from pancreatic beta cells. Clinical manifestation ranges from life-threatening hypoglycemia in neonates to mildly symptomatic hypoglycemia in early childhood, adolescence or adulthood that might sometimes be difficult to identify [1]. Up to now, mutations in nine different genes have been identified. However, in almost 50 % of CHI-cases, the genetic mechanism is still unknown, suggesting further disease-associated genes [2, 3]. CHI can be distinguished into a diffuse and focal type. Focal CHI is defined as a focal adenomatous hyperplasia of beta cells within the pancreatic tissue, which can be identified by 18F-PET/CT and completely cured by surgical removal of the focal lesion [4, 5].

In contrast, in diffuse CHI hyperplastic insulin-secreting beta cells are spread throughout the whole pancreas. Sufficient reduction of hyperinsulinemia usually requires near total pancreatectomy, which in turn implicates the risk of either persistent hypoglycemia or insulin-requiring diabetes [6, 7]. Therefore, long-term medical treatment was established during the last decades for diffuse CHI. If CHI is promptly diagnosed and adequately treated most of the children develop a normal range of cognitive, emotional and social skills. In these cases, clinical remission with termination of all drugs may occur after several years of intense conservative treatment [8, 9]. Common conservative long-term treatment options include the potassium channel opener diazoxide (which is almost ineffective in patients with defects of the K_ATP_-channel), somatostatin analogues (octreotide, lanreotide) or calcium-channel antagonists [6, 9–11]. Although these drugs are frequently used in patients with CHI, little is known about their dose, efficacy and safety. Evidence-based guidelines to manage recurrent hyperinsulinemic hypoglycemia do not exist and therefore treatment strategies remain controversial. Improved knowledge and optimization of conservative treatment is thus of major importance, especially in diffuse CHI, in which surgical cure cannot easily be achieved [7]. At present, treatment decisions are mostly based on expert opinions obtained from single-center data.

This review aims to provide more objective information collected from reports of more than 600 patients from different clinical centers. Thereby, we provide an overview of long-term conservative treatment in CHI with regard to type and duration of treatment, dose and frequency of severe as well as rare side effects that are often difficult to assess in small patient cohorts and single-center experiences.

## Methods

We searched MEDLINE (from 1947) and EMBASE (from 1988) using the OVID interface for relevant data. Search terms were combined for the disease, specific drugs and generic treatment options. Additional articles were found by cross-reading reference lists of retrieved articles. Efforts were made to translate non-english articles, which allowed us to include all relevant German, Spanish, French, Portuguese, Turkish, Danish, Polish, Chinese and Japanese articles. The latest article included for evaluation was published in June 2013.

## Results

In total 113 articles, mainly case reports and case series were evaluated with specific regard to long-term conservative treatment. The reviewed literature included a total of 1261 patients with CHI, of which 718 patients received long-term conservative treatment during their course of disease. Long-term treatment was defined as minimum duration of 6 months. Reports were also included if treatment lasted for at least two months at the time of publication but was described as being ongoing.

For 50/718 patients a specific treatment regimen was not necessary or not mentioned, another 49 patients were solely treated by frequent feedings and/or specific diets. Finally, 619 patients were medically treated and information regarding conservative treatment was available. These patients are described separately. The medication most commonly used to control hypoglycemia was diazoxide (84 %) followed by somatostatin analogues (16 %), calcium channel antagonists (4 %) and glucagon (1 %) (Table 1). For evaluation of side effects, we included all patients that did not meet our definition of long-term treatment. In order to obtain as much information as possible on very rare side effects, we furthermore discuss single patients that experienced such side effects but were treated with one of the studied medications because of a disease other than CHI (e.g. octreotide in the treatment of congenital chylothorax). These latter patients are clearly defined within the text and were not included in the analysis resulting in table 2 (Table 2).

**Table 1:** Characteristics of long-term conservative treatment in CHI: detailed description of different treatment regimen with focus on dose, combination of different drugs and duration of treatment. Patients receiving a combined therapy are listed twice

Drug	Long-term treated	Combined therapy	Dose	Course of treatment
DZX	n _total_ 521 n _previous surgery_ 16 (3 %) monotherapy n = 380 (73 %) combined therapy n = 141 (27 %)	+ diet^a^ n = 113 (79 %) + STA n = 9 (6 %) + CCA n = 2 (1 %) >2 combinations + STA + diet^a^ n = 15 (11 %) + CCA + diet^a^ n = 2 (1 %)	n _dose given_ 278 (53 %) mean: 12.5 (±4.3) mg/kg • d range: 2–60 mg/kg • d _2–3 single doses p.o. • d_	n _treatment ongoing_ 138 (26 %) duration given for n = 113/138 (82 %) mean duration: 62 months n _off medication_ 69 (13 %) duration given for n = 56/69 (81 %) mean duration: 57 months n _switched to other therapy_ 4 (1 %) partial pancreatectomy: 3/4 (75 %) STA: 1/4 (25 %) n _not mentioned; no follow-up_ 309 (59 %) n _dead_ 1 (0.2 %)
STA	n _total_ 100 OCT n = 79 (79 %) LRT n = 21 (21 %) n _previous surgery_ 17 (17 %) monotherapy n = 54 (54 %) combined therapy n = 46 (46 %)	+ diet^a^ n = 16 (35 %) + DZX n = 9 (20 %) + CCA n = 4 (9 %) + GLC n = 2 (4 %) >2 combinations + DZX + diet^a^ n = 15 (11 %)	OCT n _dose given_ 61 (77 %) mean: 14.9 (±7.5) μg/kg • d range: 2.3–50 μg/kg • d _continuously s.c. n = 28 (46 %)_ _3–6 injections s.c. • d n = 17 (28 %)_ _not specified n = 16 (26 %)_ LRT n _dose given_ 11 (52 %) mean: 67.3 (±39.8) mg • month range: 10–120 mg • month _s.c. n = 8 (38 %)_ _i.m. n = 13 (62 %)_	n _treatment ongoing_ 39 (39 %) duration given for n = 39/39 (100 %) mean duration: 35 months n _off medication_ 22 (22 %) duration given for n = 6/22 (27 %) mean duration: 49 months n _switched to other therapy_ 10 (10 %) partial pancreatectomy: 3/10 (30 %) DZX: 3/10 (30 %) gastrostomy feeding: 2/10 (20 %) DZX + CCA: 1/10 (10 %) megestrol acetate: 1/10 (10 %) n _not mentioned; no follow-up_ 29 (29 %)
CCA	n _total_ 25 NIF n = 19 (76 %) VPM ° n = 2 (8 %) AML n = 1 (4 %) not specified n = 3 (12 %) n _previous surgery_ 10 (40 %) monotherapy n = 15 (60 %) combined therapy n = 10 (40 %)	+ diet^a^ n = 2 (20 %) + DZX n = 2 (20 %) + STA n = 4 (40 %) >2 combinations + DZX + diet^a^ n = 2 (20 %)	NIF n _dose given_ 18 (95 %) mean: 0.72 (±0.36) mg/kg • d range: 0.1–2 mg/kg • d VPM n _dose given_ 2 (100 %) dose: 240 mg • d AML n _dose given_ 1 (100 %) dose: 0.1 mg/kg • d _2–4 single doses p.o. • d_	n _treatment ongoing_ 16 (64 %) duration given for n = 16/16 (100 %) mean duration: 25 months n _not mentioned; no follow-up_ 9 (36 %)
GLC	n _total_ 7 n _previous surgery_ 6 (86 %) monotherapy n = 2 (29 %) combined therapy n = 5 (71 %)	+ diet^a^ n = 3 (60 %) + STA n = 2 (40 %)	n _dose given_ 7 (100 %) given as mg/kg • d: n = 5 (71 %) mean: 0.186 (±0.14) mg/kg • d range: 0.03–0.2 mg/kg • d _continuously s.c._ given as mg • d: n = 2 (29 %) mean: 3.2 mg • d _s.c. or i.m._	n _treatment ongoing_ 1 (14 %) duration given for n = 1/1 (100 %) mean duration: 12 months n _off medication_ 3 (43 %) duration given for n = 3/3 (100 %) mean duration: 43 months n _switched to other therapy_ 2 (29 %) re-pancreatectomy: 1/2 (50 %) diet : 1/2 (50 %) n _dead_ 1 (14 %)

^a^ *Diet* frequent feeding, glucose enriched meals (dextrin, tapioca starch, ricepap), gastrostomy feeding, °*VPM* two patients with adult-onset nesidioblastosis

*DZX* diazoxide, *STA* somatostatin analogue, *CCA* calcium channel antagonists, *GLC* glucagon, *OCT* octreotide, *LRT* lanreotide (long-acting release octreotide), *NIF* nifedipine, *VPM* verapamil, *AML* amlodipine

**Table 2:** Side effects in medically treated patients with CHI: overview of side effects that occurred while on therapy with one or more of the analyzed medication. Side effects were linked to the corresponding dose that was applied. Note double entries of patients that experienced more than one side effect

Drug	n treated	Side effects	Frequency	Dose (n dose given)
DZX				mg/kg • d
	DZX treated n = 644	hypertrichosis	n = 170 (52 %)	11.6 (±2.9), range 5–30 (n = 132)
	side effects mentioned n = 325 (50 %)	fluid retention/electrolyte imbalances/edema	n = 99 (30 %)	12.3 (±2.8), range 8.5–17.5 (n = 48)
		vomiting/poor appetite	n = 40 (12 %)	13.3 (±8.2), range 10–60 (n = 39)
		bone marrow suppression	n = 11 (3 %)	10.4 (±0.46), range 10–10.8 (n = 4)
		heart failure	n = 12 (3.7 %)	16 (±6.7), range 7.5–30 (n = 7)
		anaphylactic reaction	n = 3 (0.9 %)	8.5 (n = 1)
		renal failure	n = 2 (0.6 %)	60 (n = 1)
		sinustachycardia	n = 1 (0.3 %)	up to 15 (n = 1)
		no (major) side effects *(specifically mentioned)*	n = 39 (12 %)	13.2 (±2.4), range 5–15 (n = 34)
STA				μg/kg • d or otherwise specified
	STA treated n = 355	tachyphylaxis	n = 20 (18 %)	19.5 (±7.3), range 7.1–26.3 (n = 18)
	side effects mentioned n = 111 (31 %)	GI-symptoms*	n = 23 (21 %)	17.5 (±9.5), range 6.6–27.4 (n = 20)
		(transient) impairment in growth velocity	n = 15 (14 %)	17.8 (±13.5), range 7.2–55 (n = 14)
		abnormal GH, IGF, IGF-BP *(no growth impairment)*	n = 4 (3.6 %)	25.8 (±0.72), range 25–26.3 (n = 3)
		necrotizing enterocolitis	n = 8 (7 %)	21.5 (±4.7), range 15–27 (n = 6)
		induration nodules/hematoma at injection site	n = 5 (4.5 %)	120 mg • month (n = 2)
		decline in weight	n = 3 (2.7 %)	25 (n = 1)
		asymptomatic gallstones	n = 3 (2.7 %)	33.9 (±24.8), range 6.6–55 (n = 3)
		cholestatic jaundice	n = 1 (0.9 %)	40 (n = 1)
		elevated liver enzymes	n = 3 (2.7 %)	16.8 (±11.4), range 10–30 (n = 3)
		anaphylactic reaction	n = 1 (0.9 %)	not specified
		normal growth velocity *(specifically mentioned)*	n = 52 (47 %)	19 (±8.7), range 8–55 (n = 32) 100 (±15.5) mg • month, range 90–120 (n = 6)
GLC				mg/kg • d or otherwise specified
	GLC treated n = 15	catheter obstruction	n = 9 (60 %)	0.33 (±0.23), range 0.026–0.8 (n = 9)
	side effects mentioned n = 15 (100 %)	erythema necrolyticum migrans	n = 4 (27 %)	0.3 (±0.12), range 0.22–0.46 (n = 4)
		insulin autoantibodies	n = 2 (13 %)	0.2 (n = 1), 1.4 mg • d (n = 1)
		subcutaneous infiltrates	n = 1 (7 %)	5 mg • d (n = 1)
		no side effects *(specifically mentioned)*	n = 1 (7 %)	0.21 (n = 1)

*GI-symptoms: gastrointestinal symptoms, include abdominal discomfort, fatty stock, diarrhea, unwillingness to eat; °hematoma was notified three times out of 70 i.m. injections in a total of ten patients treated with lanreotide

*DZX* diazoxide, *STA* somatostatin analogue, *GLC* glucagon, *GH* growth hormone, *IGF* insulin-like growth factor, *IGF-BP* insulin-like growth factor binding protein

### Dietary treatment

In 49 patients a “sufficient control” of hypoglycemia was achieved with nutritional treatment. Importantly, the definition of “sufficient control” might vary greatly among the different hyperinsulinism centers further stressing the necessity of establishing a standardized fasting tolerance test to better compare the response to dietary or medical treatment in between the different clinical centers.

Almost half of these patients (49 %) were fed frequently with glucose-enriched meals. Another eight patients (16 %) additionally required raw cornstarch and also glucocorticoids for a sufficient control of hypoglycemia [12]. Seventeen patients (35 %) were treated with a leucine-restricted diet, of which all except two patients suffered from the Hyperinsulinism-Hyperammonemia Syndrome (HI/HA) caused by a mutation of the GLUD1 gene. The other two patients treated with a leucine-restricted diet were subsequently diagnosed as carrying a dominantly inherited SUR1 mutation. Both of them developed mental retardation due to recurrent seizures [13].

## Discussion

The purpose of this study was to assemble information on medical treatment of CHI with specific regard to type and duration of treatment, dose, as well as side effects. Since we collected as much information as available, including case reports in various languages from different clinical centers worldwide, the strength of the evidence has to be critically assessed. Our results are biased by the fact that publication practices tend to favor positive treatment responses and that very rare or severe side effects are more likely to be published. However, we believe that with a more rigorous approach with regard to the quality of data, a lot of important information would have been lost. The lack of objective information on long-term medical treatment in CHI, in particular with respect to the heterogeneity of the condition itself is a general dilemma for the treatment of patients with CHI and appropriate counseling of their parents. We are therefore convinced that the careful recapitulation of what has been reported for a large cohort of patients might help to improve the knowledge and decisions on long-term treatment of CHI even with respect to the weakness that published data are biased and the lack of a more critical approach to the evaluation of the reports.

### Diazoxide

Diazoxide is a K_ATP_-channel opener and the only drug approved by the Food and Drug Administration (FDA) for long-term treatment of hyperinsulinemic hypoglycemia. However, many patients are diazoxide resistant due to mutations in the two genes encoding the K_ATP_-channel of the pancreatic beta cell (*ABCC8/KCNJ11*) [14] In total 521 patients were treated long-term with diazoxide, either alone (73 %) or in combination with other drugs or frequent feeds/specific diets (27 %). For evaluation of side effects we included reports on short-term use of diazoxide, thus evaluating a total number of 644 cases of diazoxide application.

#### Hypertrichosis

The most frequent side effect was a mild to severe hypertrichosis, which is thought to appear dose dependent in every patient. Of note, minoxidil, another potassium channel opener has been used as hair-regrowing treatment for more than 20 years [15]. In the evaluated patients hypertrichosis was reported in only 52 % of children and seemed to be dose-independent, e.g. it occurred in a patient treated with a dose as low as 6 mg/kg ⋅ d [16] but it was not present in several children treated with a dose of 15 mg/kg ⋅ d [17]. However, this may be due to underreporting, as hypertrichosis is often considered to be of minor clinical importance. In contrast, for treated patients hypertrichosis might imply stigmatization, leading to psychological distress and ultimately withdrawal of diazoxide, which was reported for two patients [12, 18].

Theoretically, topical K_ATP_-channel blocker, such as tolbutamide may be able to reduce hair growth, but is has not yet been evaluated systematically for this indication [19].

#### Fluid retention

Less frequently, fluid retention and electrolyte imbalances were described without further specification (30 %). Diazoxide is a benzothiadiazine derivate and decreases renal sodium-, chloride- as well as bicarbonate excretion without affecting potassium excretion leading to an expansion of extracellular fluid volume [20–22]. This appears to be opposite to other benozothiadiazines, which can antagonize water and sodium retention during diazoxide treatment [21]. Therefore, diazoxide is often combined with hydrochlorothiazide (HCT). Given the low rate of fluid imbalances with diazoxide, a combined treatment is not always necessary. However, under certain conditions it seems reasonable to start with a low dose of diazoxide and to gradually increase the dose over the first few days of treatment, e.g. in newborns with a high glucose infusion rate (fluid overload) and/or cardiac risk factors. Alternatively, if one whishes to start with a higher dose of diazoxide, a combined treatment with diazoxide and HCT can be considered even though signs of fluid retention are not yet present. The latter approach allows a more rapid transition to alternative treatment in case of diazoxide unresponsiveness.

#### Gastrointestinal symptoms (GI-symptoms)

Gastrointestinal (GI) symptoms, such as poor appetite, nausea and vomiting occurred in 12 % of patients. Though not generally being severe, these can complicate treatment in CHI especially if additional dietary treatment is needed.

Severe side effects of diazoxide, i.e. heart failure (3.7 %), renal failure and petechiae (0.6 %) or anaphylactic reactions (0.9 %) and laboratory parameters of bone marrow suppression (4 %) were rarely seen. In many of these cases, a causal relation between diazoxide and these side effects might be disputable.

#### Cardiotoxicity

Cardiotoxicity of diazoxide has been questioned by various authors. Cardiomyopathy in CHI resembles cardiomyopathy seen in children of diabetic mothers, suggesting hyperinsulinemia and not diazoxide treatment as the underlying cause [23, 24]. However, the temporal relation between cessation of diazoxide treatment and resolution of cardiac abnormalities despite persistent hyperinsulinemia as described for some patients suggests a causative role of diazoxide [25]. Cardiac failure might be indirectly caused by diazoxide through water and salt retention and thus fluid overload [26] or directly by inducing an increase in pulmonary blood flow resulting in an increased right ventricular work as shown in patients with primary pulmonary hypertension [27].

Finally, in cardiomycoytes Kir6.2 is integral in the make-up of myocellular K_ATP_-channels and K_ATP_-channel malfunction has been implicated in the development and progression of heart disease [24, 28]. Taken together, heart failure could ultimately result from 1) hyperinsulinemia itself, 2) diazoxide treatment, most probably as a secondary complication due to fluid overload or due to a direct hemodynamic effect on pulmonary blood flow or 3) an underlying K_ATP_-channel mutation.

#### Renal failure

Two patients developed renal failure under treatment with diazoxide (<1 %). Both suffered from oliguria with elevated urea- or creatinin-levels and additionally developed petechiae, either generalized or as quick transient petechial rash on the back of the feet while coagulation profile and/or platelet count was normal [29, 30]. These both patients had additional problems, i.e. fluid retention and cardiac decompensation or infection. Therefore it seems more likely that renal failure was indirectly caused rather than being due to nephrotoxicity of diazoxide.

#### Anaphylactic reaction and bone marrow suppression

Anaphylactic reactions were described for three patients (<1 %), requiring reduction of diazoxide dose from 600 mg ⋅ d to 350 mg ⋅ d in an adult patient with adult-onset nesidioblastosis with facial flushing, edema and a slight decrease in blood pressure from 140/78 mmHg to 120/70 mmHg [31] or cessation of diazoxide treatment in two patients with recurrent rash or severe anaphylactic reaction (not further specified), respectively [32]. Bone marrow suppression (leucopenia, isolated neutropenia or thrombocytopenia) occurred in 11 patients (3 %) with CHI and in two further patients of which one additionally suffered from pulmonary hypertension and heart failure, whereas in the other hyperinsulinism was due to Münchausen syndrome by proxy [33, 34]. In these two patients neutropenia resolved when diazoxide was ceased. In another two patients diazoxide treatment was ceased due to neutropenia [12, 35], in the others outcome was not mentioned. Severe infection was not mentioned for any patient with leucopenia suggesting that this was not the case.

In summary, diazoxide is still the first line drug in the treatment of hyperinsulinism. It is easy to apply and generally well tolerated. Common side effects are usually not severe. A causal relation between diazoxide and severe side effects remains doubtful as there are only very few cases reported and detailed information on the course of treatment is limited.

### Octreotide

The hypothalamic release-inhibiting hormone somatostatin is a cyclic polypeptide that is found throughout the nervous and gastrointestinal system. It binds to specific membrane receptors called somatostatin receptors (SSTR1-SSTR5) [36]. In the pancreas, somatostatin has inhibitory effects on the release of glucagon and insulin from the pancreatic islet cells and suppresses the incretin glucagon-like-peptide 1 (GLP-1) [37]. Other actions include inhibition of gastrointestinal motility, gallbladder contractility and splanchnic blood flow [38]. Early somatostatin infusion tests in infants and children with hyperinsulinism confirmed its efficacy in CHI [39, 40]. However, clinical use of somatostatin was restricted due to its half-life of less than three minutes [36]. Therefore, various synthetic analogues of somatostatin were tested for its efficacy in CHI [41–43]. Octreotide and lanreotide have a prolonged half-life of 90 minutes (octreotide) and 23–30 days (lanreotide), respectively, when administered by subcutaneous (s.c.) or intramuscular (i.m., lanreotide) injections (FDA). Though not officially approved for this indication it is now common to use somatostatin analogues in the treatment of hyperinsulinism (off-label use). The use of lanreotide as an alternative to octreotide pump treatment is relatively new but has already been proven successful in children with CHI [42, 43]. Recently, a new somatostatin analogue preferably binding to SSTR5 (pasireotide), was granted marketing for treatment of cushing’s disease [44]. In future, pasireotide could also be of use in the treatment of CHI.

In total, 100 patients were treated long-term with octreotide (79 %) or lanreotide (21 %), either alone (54 %) or in combination with other drugs or frequent feeds/specific diets (46 %). For evaluation of side effects we again included reports on short-term use of somatostatin analogues (in total 355 cases).

#### Tachyphylaxis

An important aspect of somatostatin analogues in the treatment of CHI is the loss of effect during prolonged treatment (tachyphylaxis), which usually occurs within the first days or weeks of treatment. Tachyphylaxis occurred in a total number of 20 patients with CHI (18 %). Interestingly, tachyphylaxis is generally not observed in patients treated with octreotide for acromegaly. Several authors propose tachyphylaxis to be caused by gastrointestinal adaption, e.g. through tissue-specific desensitization or down-regulation of somatostatin receptors while SSTR expressed in pituitary tumours might have a different turn-over rate and thus escape local adaption [36–38].

#### Gastrointestinal symptoms, gallstones, hepatitis

Most adverse effects such as abdominal pain or stool abnormalities were mild and resolved spontaneously within a few weeks under ongoing treatment. Further gastrointestinal side effects include gallstones and necrotizing enterocolitis (NEC), which can be partially explained by the action profile of natural somatostatins. Of the evaluated patients only four of them were reported to have gallstones (3.6 %) [42, 43, 45, 46], of which three were asymptomatic and octreotide/lanreotide treatment was continued in combination with ursodeoxycholic acid. However, the incidence of asymptomatic gallstones might be higher. In acromegaly, up to 20–30 % of patients treated with octreotide develop gallstones [36] consistent with a recent report of 28 patients with CHI on octreotide treatment in which gallbladder pathology was detected in 32 % of children [10]. Since the recognition or prevention of asymptomatic gallstones might help to prevent complications, routine ultrasound once a year or prophylactic treatment with ursodeoxycholic acid during treatment with somatostatin analogues seems feasible. In three patients liver enzymes increased while on treatment with octreotide, which returned to normal levels in all when treatment was ceased [47–49]. Interestingly, in two of these, octreotide-induced hepatitis was associated with either a high dose (up to 40 μg/kg ⋅ d) or with a gradual increase in dose from 5 to 10 μg/kg ⋅ d. This finding stresses the importance of dose in the development of octreotide-induced hepatitis and warrants routine examination of liver function tests particularly when high doses are given or when dosing is increased [47].

#### Necrotizing enterocolitis (NEC)

The most severe side effect that is discussed to be associated with somatostatin analogues is NEC. A causal relation between octreotide and NEC is not yet proven, although the substantial reduction in splanchnic blood flow induced by octreotide suggests a causative role [50].

Four of eight children that developed NEC while on treatment with octreotide clearly had other risk factors for NEC: severe cardiomyopathy (*n* = 1) [51], congestive heart failure due to excessive fluid overload (*n* = 2) [52, 53], and a patent ductus arteriosus (PDA) (*n* = 1) [50]. Remarkably, in one of these patients the condition quickly improved upon adequate treatment even though octreotide was increased two-fold [50]. Severity of NEC ranged from bloody stools to death (*n* = 2). Another patient additionally received nifedipine and metoclopramide, which could as well have contributed to the development of bowel gangrene [50]. Of the remaining three patients, two had no additional extrinsic risk factors and for one patient detailed clinical history was not provided [50]. Six of the patients that developed NEC were full-term neonates, for two patients gestational age was not given. All children received octreotide within the first month of life, for one child age was not given. The occurrence of NEC may be associated with the applied dose, as a dose-dependent reduction in splanchnic blood flow was shown in rats [54]. All children that developed NEC received a dose ≥15 μg/kg ⋅ d and the mean dose of 21.5(±4.7) μg/kg ⋅ d was remarkably higher than the mean dose in patients without NEC (14.9 μg/kg ⋅ d).

An additional three neonates with NEC were treated with octreotide for other indications. One of these patients was born premature and had a PDA [55], the other two both have had extensive cardiac surgery previous to the development of NEC with documented distal aortic ischemia in one patient [50, 56]. An adult patient treated with octreotide for another indication already had pre-existing gastrointestinal symptoms with abdominal distension and steatorrhoea, which worsened under treatment with octreotide.

Taken together, in most of the cases reported octreotide is merely a risk factor among others. However, all children received octreotide within the first months of life, all of them developed NEC within the first days or weeks of treatment and all except one were treated with a dose ≥15 μg/kg ⋅ d.

#### Growth deceleration

Since somatostatin inhibits growth hormone secretion, treatment with somatostatin analogues might compromise growth velocity. Mild growth deceleration was observed in 15 patients (13.5 %) [42, 45, 57–60]. Of these, eight patients caught up after treatment was discontinued, dose decreased or even under ongoing treatment. In these patients, height normalized appropriate for parental target height. Only five patients (4.5 %) continued to grow below their target range [45, 57, 59, 60]. In one patient outcome was not specified, another patient was still on treatment but height remained only 0.6 SD below the expected height. For 52 patients (47 %) it was particularly mentioned that they grew normal while on long-term treatment with somatostatin analogues. In summary, the risk of persistent growth deceleration and inappropriate target height is very low. Nevertheless, growth should be closely monitored in all patients during and after somatostatin analogue treatment.

### Calcium channel antagonists

Calcium channel antagonists such as nifedipine or amlodipine are approved for treatment of hypertension and angina pectoris. Their use in treatment of CHI is relatively new. They inhibit the transmembrane influx of calcium ions into the pancreatic beta cells, known to be an essential trigger for insulin secretion [11]. However, most hyperinsulinism centers do not consider these drugs as indicated because of the lack of effectiveness. Therefore, in the treatment of CHI, calcium channel antagonists are merely used as a third-line drug, e.g. as an add-on treatment, in partial diazoxide/octreotide resistance, and/or following partial pancreatectomy [16, 61, 62]. In fact, 40 % of the evaluated patients had partial to sub-total pancreatectomy previous to treatment with calcium channel antagonist, compared to 3 % and 17 % of patients treated with diazoxide and somatostatin analogues, respectively. Possible side effects include hypotension, peripheral edema, headache, flushing, dizziness and nausea (FDA). In CHI, calcium channel antagonists were well tolerated, which might be related to the relatively low dose: mean dose was 0.72 (±0.36) mg/kg ⋅ d (range 0.1-2 mg/kg ⋅ d) in patients treated with nifedipine and 0.1 mg/kg ⋅ d in two patients treated with amlodipine, respectively, which is in the lower range of the recommended dose for the treatment of hypertension in pediatric patients (nifedipine: 0.25-3 mg/kg ⋅ d; amlodipine: 0.05-0.5 mg/kg ⋅ d) [63].

In summary, while calcium channel antagonists are safe in the treatment of hyperinsulinism, these agents do not seem to sufficiently restore normoglycemia and are therefore in our opinion not indicated as first line drugs in the treatment of CHI.

### Glucagon

Glucagon is a polypeptide hormone physiologically secreted by alpha cells of the pancreatic islets to promote hepatic glycogenolysis and gluconeogenesis and thus directly increases blood glucose levels [6]. Initially, intravenous glucagon infusion is often used in CHI for several days or weeks to stabilize blood glucose levels, however, its long-term use is limited due to its short half-life and the need for parenteral administration.

Only 7 patients were treated long-term with glucagon, either continuously s.c. or by frequent injections s.c. or i.m [64–68]. Importantly, in all but one patient glucagon was used in combination with other drugs or following subtotal pancreatectomy. Continuous s.c. glucagon infusion is frequently complicated by catheter obstructions occurring daily or 2–3 times per week [65]. A stabilized formulation of glucagon was successfully administered in three children until surgery could be performed resulting in less problems regarding catheter blockage [65]. Two of these patients and two additional patients reported by Wald et al. developed erythema necrolyticum migrans [65, 69]. This might be explained by an increased bioavailability of the stabilized formulation of glucagon and by the relatively high dose (up to 0.22 mg/kg ⋅ d intravenous (i.v.) and 0.26 mg/kg ⋅ d s.c.) administered in the two patients reported by Wald et al., respectively. In these four patients glucagon was administered s.c. over periods of up to 3.5 months (*n* = 3) or i.v. as short-term management of hypoglycemia (*n* = 1). In all patients lesions resolved without scarring. Less severe side effects included subcutaneous infiltrates at the site of injection (*n* = 1) [67] and the development of insulin autoantibodies (*n* = 2), possibly due to the amount of insulin in the glucagon preparation (up to 0.02 %) [64, 68].

In summary, glucagon is very helpful for the treatment of hypoglycemia within the first weeks of life and it might be beneficial following pancreatectomy and as an add-on treatment, particularly in combination with a somatostatin analogue to replace the octreotide/lanreotide-induced suppression of endogenous glucagon secretion. However, long-term treatment is still limited by the lack of a suitable preparation for continuous s.c. infusion.

### Outlook

Although there is a broad spectrum of drugs available for children with CHI, a considerable number of patients do not show sufficient response to existing medical treatment. Therefore, efforts are made to provide these patients with alternative drugs. Recently, exendin-(9–39) a specific GLP-1 receptor antagonist was shown to elevate fasting blood glucose levels in nine individuals with K_ATP_-CHI [70]. Furthermore, the mammalian target of rapamycin (mTor) inhibitor sirolimus has recently been proven successful in four infants with severe hyperinsulinemic hypoglycemia unresponsive to diazoxide [71]. Another promising approach is the development of new somatostatin analogues, such as pasireotide, with modified binding profiles and thus possibly a stronger inhibitory effect on insulin secretion [44].

## Conclusion

Despite increasing knowledge on etiology, data available on long-term conservative treatment of CHI are still poor. There exists an urgent need for prospective studies or registers to better evaluate safety and efficacy of drug treatment in CHI. This work serves as a basis to reconsider treatment strategies and provides information especially on a rough frequency and impact of rare side effects of drugs given to patients with CHI.

Recommendations based on our analysis:

- Diazoxide
  - it is important to screen for signs of fluid retention; to monitor blood gases, sodium, chloride, bicarbonate and potassium level, in particular within the first weeks of treatment and when glucose infusion rate is high; when indicated hydrochlorothiazide should be added.
  - in neonates cardiac monitoring (echocardiography) should be performed.
- Somatostatin analogues
  - somatostatin analogues should be avoided in neonates, particularly if additional risk factors for NEC are present; if not possible, an initial dose should not be higher than 15 μg/kg ⋅ d and excessive fluid overload must be prevented, as this may trigger the risk of NEC; treatment must immediately be stopped once any sign of NEC is present.
  - routine abdominal ultrasound should be performed prior to and every 12 months after initiation of treatment; if gallstones are present ursodeoxycholic acid should be added.
  - growth curves must be monitored; dose should only be decreased if marked growth deceleration becomes evident, as in most of the children the decrease in growth velocity is transient.
  - liver function must be monitored, particularly if using a high dose or when dosing is increased.
  - lanreotide should not be used in neonates as severe side effects might require immediate discontinuation of treatment.
